# Association between proteinuria changes and colorectal cancer incidence: evidence from a nationwide cohort study

**DOI:** 10.1186/s12876-025-03935-7

**Published:** 2025-05-21

**Authors:** Soo Young Oh, Kyung-Do Han, Ga Yoon Ku, Won-Kyung Kang

**Affiliations:** 1 Department of Surgery, Yeouido St. Mary’s Hospital, College of Medicine, The Catholic University of Korea, 10, 63-ro, Yeongdeungpo-gu, Seoul, Republic of Korea; 2 Department of Statistics and Actuarial Science, Soongsil University, Seoul, Republic of Korea; 3 Division of Colon and Rectal Surgery, Department of Surgery, University of Ulsan College of Medicine, Asan Medical Center, Seoul, South Korea

**Keywords:** Colorectal cancer, Proteinuria, Cancer risk factors, Cancer incidence, Nationwide study

## Abstract

**Background:**

The presence of excess proteins in the urine, known as proteinuria, has been linked to various health conditions, including chronic kidney disease and cancer. Emerging evidence suggests an association between proteinuria and colorectal cancer, a leading global cause of cancer-related morbidity and mortality. However, the impact of changes in proteinuria status on colorectal cancer risk remains unclear. Understanding this relationship may identify proteinuria as a modifiable risk factor for colorectal cancer prevention.

**Methods:**

This retrospective cohort study analyzed data from 3,043,138 adults aged over 19 years who participated in biennial health screenings by the South Korean National Health Insurance Service in 2010 and 2012. Participants were classified into four groups based on changes in proteinuria status: no proteinuria, newly developed proteinuria, recovered proteinuria, and persistent proteinuria. Proteinuria was measured using dipstick urinalysis, and colorectal cancer diagnoses were identified using medical records. Cox proportional hazards models adjusted for age, sex, body mass index, lifestyle behaviors, and other confounders were used to estimate cancer risk.

**Results:**

Over a median follow-up period of 9.19 years, 36,846 participants (1.2%) developed colorectal cancer. After adjusting for multiple confounding factors, including age, sex, lifestyle behaviors, medication use, diabetes, hypertension, dyslipidemia, and chronic kidney disease, the persistent proteinuria group demonstrated a significantly higher risk of colorectal cancer compared with the proteinuria-free group (adjusted hazard ratio [aHR], 1.27; 95% CI, 1.13–1.42). Additionally, greater severity of proteinuria was associated with progressively increased colorectal cancer risk (aHR for overt proteinuria [+ 2 to + 4], 1.17; 95% CI, 1.05–1.29).

**Conclusions:**

Changes in proteinuria status are significantly associated with colorectal cancer risk. Persistent proteinuria poses the highest risk, while transient proteinuria also elevates risk compared to individuals without proteinuria. Regular monitoring and management of proteinuria could potentially be beneficial in identifying individuals at higher colorectal cancer risk, suggesting its possible role as an indicator for targeted prevention strategies. However, further research, including randomized controlled trials, is necessary to confirm any causal relationship.

**Supplementary Information:**

The online version contains supplementary material available at 10.1186/s12876-025-03935-7.

## Background

Proteinuria (PU), a condition characterized by the presence of excess proteins in the urine, has been implicated in various health issues, including chronic kidney disease (CKD) and cardiovascular events [1–3]. Emerging evidence indicates a potential link between PU and increased risk of malignancies, especially colorectal cancer (CRC), one of the most prevalent cancers globally [4–6]. Despite advances in screening and therapeutic strategies, substantial public health challenges remain due to its high incidence and mortality and efforts are ongoing to increase our understanding of CRC, including identification of causative factors.

The pathophysiological mechanisms linking PU and cancer are complex and multifaceted. PU often reflects underlying microvascular damage and increased glomerular permeability, which may be driven by systemic inflammation and cytokine release associated with malignancies ​​ [7, 8]. This inflammatory milieu can contribute to both the progression of renal disease and the development of cancer, which may exacerbate the outcomes for patient​s. Albuminuria, a specific type of PU, was shown prevalent in patients with various cancers, including lung, breast, renal cell, and gastrointestinal including CRC [7, 9–16]. For CRC in particular, patients with early-stage CRC who present with PU were found to face higher risk of cancer-specific mortality compared with subjects without PU [17]. The potential of PU as a prognostic biomarker in cancer management highlights the need for its early detection and monitoring in at-risk populations.

To address this gap, this study aims to elucidate the effects of changes and persistence in PU status on CRC risk, accounting for the dynamic nature of PU and its potential reversibility. The purpose of the study is to provide new insights of PU as a modifiable risk factor for CRC and inform clinical practicesdirected at reducing cancer risk through effective PU management.

## Methods

### Data collection

This retrospective, population-based cohort study was conducted using the South Korean population database provided by the National Health Insurance Service (NHIS). The Korean NHIS provides national health screenings every 2 years for all insured persons at no charge, covering 97% of the total population. The participation rate of the target population is approximately 75% [18]. The NHIS database includes anthropometric measurements, medical history, laboratory tests, and health-related behaviors. Extensive data regarding clinical information, including demographic data, history of medical treatments (hospital admissions, procedures, and prescriptions), and diagnostic codes can be obtained using the NHIS data based in the International Classification of Diseases, 10th Revision, Clinical Modification (ICD-10-CM) codes.

The Institutional Review Board (IRB) of The Catholic University of Korea approved this study (IRB No. SC24ZISE0055). The requirement for written informed consent was waived because the NHIS data set was obtained anonymously with strict confidentiality guidelines. This study was conducted in accordance with the principles of the Declaration of Helsinki.

### Study population

**Fig. 1:**
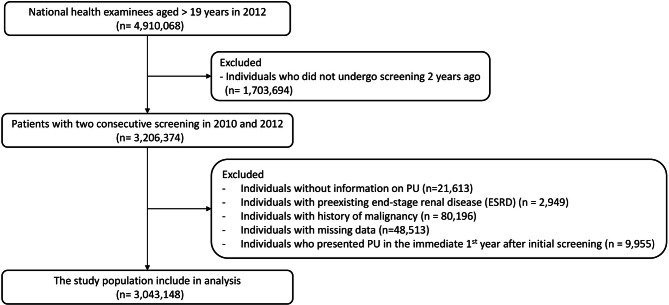
Flowchart of the study population selection process. This flowchart illustrates the inclusion and exclusion criteria used to identify the final cohort of participants from the initial pool of individuals who underwent health screenings. It highlights the stepwise exclusions based on missing data, pre-existing conditions, and other eligibility factors, leading to the final cohort of 3,043,138 participants

The enrollment process of the cohort is depicted in Fig. 1. A total of 4,910,068 participants > 19 years of age who underwent the national health examination in 2012 were initially selected. Among subjects, 3,206,374 participants who had also undergone the health screening in 2010 were selected. Participants without information on PU (*n* = 21,613), with preexisting end-stage renal disease (ESRD; *n* = 2,949), with a history of malignancy (*n* = 80,196), or with missing variables (*n* = 48,513) were excluded from the study. In addition, participants diagnosed with CRC within the first year immediately following the second screening (*n* = 9,955) were excluded from the cohort to minimize potential reverse causality. Finally, a total of 3,043,138 participants were followed up until the development of CRC, death, or December 2022, the earliest of which occurred.

### Definitions of clinical variables

The NHIS health checkup includes demographic characteristics such as age and sex, laboratory tests, and self-reported questionnaires used to collect health-related behavior including smoking status, alcohol consumption, and physical activity. Smoking status was surveyed based on at the time of the study as nonsmoker, ex-smoker, or current smoker. Alcohol consumption was defined as none, mild (< 30 g/d), or heavy (≥ 30 g/d) based on the frequency of intake per week and the amount consumed per drinking episode. Regular exercise was defined as exercise three or more times of intense 20-minute workouts per week or 5 or more times of moderate 30-minute workouts per week. Obesity was classified as body mass index (BMI) ≥ 25 kg/m² based on Korean criteria [19]. Trained examiners measured height, weight, and waist circumference (WC) [20]. Blood pressures were measured at least 5 min of rest and blood samples were collected after an overnight fast lasting at least 8 h.

Diabetes was identified based on a fasting glucose level ≥ 126 mg/dL or at least one annual claim for a prescription of antidiabetic medications (either oral or injectable) under ICD-10-CM codes E11–E14. Hypertension was defined based on a systolic blood pressure ≥ 140 mmHg, diastolic blood pressure ≥ 90 mmHg, or prescription of antihypertensive medications under ICD-10-CM codes I10–I13 and I15. Dyslipidemia was diagnosed either based on a total cholesterol level ≥ 240 mg/dL or prescriptions for lipid-lowering medications under ICD-10-CM code E78. Patients with CKD were defined as subjects with an eGFR < 60 mL/min/1.73 m² calculated using the MDRD equation. Patient use of renin-angiotensin system (RAS) inhibitors, commonly prescribed to manage PU by reducing glomerular pressure and minimizing protein leakage into urine, was identified by reviewing claims for at least one annual prescription [21].

### Proteinuria and outcome analysis

PU was assessed using dipstick urinalysis. Urine samples were collected from random spot specimens obtained early in the morning after an overnight fast. The dipstick results were interpreted using a semi-quantitative color scale, ranging from negative to trace (±), 1+, 2+, 3+, or 4+. Dipstick PU results were classified into three categories: no PU (−), trace (±), and overt PU (1 + or higher). The same dipstick method was used for both the 2010 and 2012 NHID national screenings to measure PU, allowing for evaluation of changes in dipstick PU over the 2-year period for each participant.

Study participants were classified into four groups based on changes in their PU status by the presence or absence of PU at first screening (S1) and second screening (S2). Individuals without PU at S1 were categorized into either the PU-free group (no PU at both S1 and S2) or the PU-developed group (no PU at S1 but presence of PU at S2). Participants with PU at S1 were categorized into the PU-persistent group (PU present at both S1 and S2) or the PU-recovered group (PU present at S1 but absent at S2) based on the change in PU status confirmed at S2.

The primary outcome was the newly diagnosed CRC identified based on hospitalization with the respective ICD-10-CM codes. CRC is classified using ICD-10-CM codes C18–C20, with specific codes for different tumor locations: C18.0–C18.4 for right-sided colon cancer, C18.5–C18.7 and C19.0 for left-sided colon cancer, and C20.0 for rectal cancer, along with the cancer registration code (V193). Since 2006, the NHIS policy enhanced the health coverage for cancer, and to qualify for a reduced copayment rate of 5% for cancer-related tests and treatments, physicians and medical institutions are required to certify cancer diagnoses using a special reimbursement code (V193). This code ensures that all patients with a confirmed cancer diagnosis are included in the national NHIS registry. In the subgroup analysis based on tumor location, cases of malignant neoplasms in overlapping colon sites (C18.8) and unspecified colon cancer (C18.9) were excluded because they were not specific to a single location.

### Statistical analysis

The baseline characteristics were compared using one-way analysis of variance for continuous variables and chi-square tests for categorical variables. CRC incidence rates were determined by dividing the number of cases by 1,000 person-years. Kaplan-Meier curves were used to plot the cumulative incidence probabilities of CRC, which were then compared using the log-rank test. To evaluate hazard ratios (HRs) and 95% confidence intervals (CIs) for the associations between baseline PU, changes in PU, and CRC risk, multivariable Cox proportional hazards regression models were applied. In addition, the relationships among baseline PU components, changes in these components, and the risk of CRC based on the site of CRC were examined. Model 1 was unadjusted, and Model 2 was adjusted for age and sex. Model 3 included further adjustments for smoking status, alcohol consumption, physical activity, and BMI. Model 4 included additional adjustments for the use of RAS inhibitors. Model 5 was additionally adjusted for diabetes, hypertension, and dyslipidemia. Model 6 included further adjustment for chronic kidney disease. Subgroup analyses were conducted to explore the associations between the severity of PU status and CRC risk. Sensitivity analysis was performed by excluding participants diagnosed with diabetes mellitus and chronic kidney disease to evaluate potential residual confounding effects of these comorbid conditions.

Model performance was assessed using Harrell’s concordance index (C-index) to evaluate the predictive discrimination of our Cox proportional hazards models. Model calibration was assessed using calibration plots comparing predicted probabilities with observed outcomes for the final adjusted model (Model 6). Internal validation was conducted using bootstrap resampling (limited to 50 iterations due to computational constraints arising from the large dataset) to compute the optimism-corrected C-index, assessing potential overfitting and the generalizability of the model. The proportional hazards assumption for each variable was examined using Schoenfeld residual tests. All statistical tests were two-sided with statistical significance set at *p* < 0.05. The statistical analyses were performed using SAS software (version 9.4; SAS Institute, Cary, NC, USA).

## Results

### Baseline characteristics

**Fig. 2:**
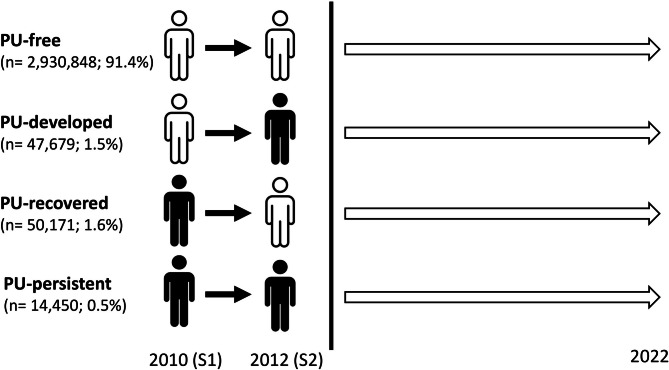
Classification of participants based on proteinuria status across two screening periods (2010 and 2012). Participants were categorized into four groups based on changes in proteinuria (PU) status: PU-free (No → No), PU-developed (No → Yes), PU-recovered (Yes → No), and PU-persistent (Yes → Yes). The diagram depicts the transitions in PU status and the respective group sizes. PU-free individuals are represented in white, while individuals with proteinuria are represented in black

A total of 3,043,148 participants were included in the analysis after excluding individuals with missing data (Fig. 1). Participants were classified based on their PU status across two screening periods into four groups (Fig. 2): PU-free (No → No, 91.4%), PU-developed (No → Yes, 1.5%), PU-recovered (Yes → No, 1.6%), and PU-persistent (Yes → Yes, 0.5%).

The baseline characteristics shown in Table 1, including age, gender distribution, and initial health metrics, were compared across the groups. Significant differences were observed in the baseline characteristics among the groups. Notably, subjects with persistent PU were the oldest (55.88 ± 13.02 years) and had the highest proportion of males (67.65%). This group also had the highest proportion of current smokers and heavy drinkers. Furthermore, the PU-persistent group had the highest prevalence of diabetes, hypertension, dyslipidemia, and CKD.

**Table 1:** Baseline characteristics of the study population

	Proteinuria Change				*p*-value
	Free	Developed	Recovered	Persistent	
	(*n* = 2,930,848)	(*n* = 47,679)	(*n* = 50,171)	(*n* = 14,450)	
Age, years	49.19 ± 13.24	52.64 ± 14.02	52.48 ± 13.77	55.88 ± 13.02	< 0.0001
Age group					< 0.0001
< 40	752,228 (25.67)	8971 (18.82)	9327 (18.59)	1622 (11.22)	
40–64	1,776,451 (60.61)	28,420 (59.61)	30,603 (61)	8922 (61.74)	
≥ 65	402,169 (13.72)	10,288 (21.58)	10,241 (20.41)	3906 (27.03)	
Sex, male	1,656,206 (56.51)	27,042 (56.72)	27,275 (54.36)	9776 (67.65)	< 0.0001
Smoking					< 0.0001
Nonsmoker	1,731,076 (59.06)	27,795 (58.3)	30,174 (60.14)	7491 (51.84)	
Ex-smoker	504,327 (17.21)	8777 (18.41)	8866 (17.67)	3352 (23.2)	
Current smoker	695,445 (23.73)	11,107 (23.3)	11,131 (22.19)	3607 (24.96)	
Drinking					< 0.0001
Non	1,477,279 (50.4)	25,168 (52.79)	26,926 (53.67)	7880 (54.53)	
Mild	1,246,460 (42.53)	18,370 (38.53)	19,334 (38.54)	5306 (36.72)	
Heavy	207,109 (7.07)	4141 (8.69)	3911 (7.8)	1264 (8.75)	
Regular exercise	611,037 (20.85)	9949 (20.87)	10,717 (21.36)	3114 (21.55)	0.01
Diabetes	280,645 (9.58)	11,834 (24.82)	11,384 (22.69)	6089 (42.14)	< 0.0001
Hypertension	817,815 (27.9)	22,616 (47.43)	23,503 (46.85)	10,466 (72.43)	< 0.0001
Dyslipidemia	632,460 (21.58)	15,562 (32.64)	15,739 (31.37)	7149 (49.47)	< 0.0001
Chronic Kidney Disease	99,396 (3.39)	5156 (10.81)	4474 (8.92)	4140 (28.65)	< 0.0001
Obesity	985,410 (33.62)	19,595 (41.1)	20,653 (41.17)	7206 (49.87)	< 0.0001
RAS Inhibitor use	511,730 (17.46)	15,514 (32.54)	17,431 (34.74)	8263 (57.18)	< 0.0001
Height, cm	164.28 ± 9.24	163.48 ± 9.19	163.3 ± 9.14	164.11 ± 8.85	< 0.0001
Weight, kg	64.56 ± 11.75	65.42 ± 12.61	65.35 ± 12.35	68.01 ± 12.88	< 0.0001
BMI, kg/m2	23.82 ± 3.17	24.37 ± 3.57	24.4 ± 3.52	25.14 ± 3.66	< 0.0001
Waist circumference, cm	80.53 ± 9	82.55 ± 9.88	82.32 ± 9.77	85.4 ± 9.68	< 0.0001
Systolic blood pressure, mmHg	122.04 ± 14.46	126.25 ± 16.89	125.1 ± 15.79	131.55 ± 17.18	< 0.0001
Diastolic blood pressure, mmHg	76.19 ± 9.79	78.34 ± 11.03	77.49 ± 10.41	80.48 ± 11.18	< 0.0001
Fasting glucose, mg/dL	97.41 ± 21.33	108.93 ± 37.5	105.09 ± 33.01	119.61 ± 47.24	< 0.0001
Total cholesterol, mg/dL	195.6 ± 36.15	197.77 ± 40.17	194.35 ± 38.37	197.62 ± 44.39	< 0.0001
HDL-C, mg/dL	55.2 ± 17.32	54.55 ± 18.31	54.08 ± 17.01	51.29 ± 14.46	< 0.0001
LDL-C, mg/dL	114.94 ± 33.75	115.07 ± 37.57	112.56 ± 35.39	113.04 ± 39.11	< 0.0001
Triglyceride*, mg/dL	110.84 (110.77, 110.92)	120.57 (119.93, 121.22)	119.41 (118.8, 120.01)	144.67 (143.31, 146.04)	< 0.0001

* Geometric Mean (95% CI)

When comparing the PU-developed and PU-recovered groups, the PU-recovered group showed a higher proportion of non-smokers and non-drinkers, with a smaller proportion of heavy drinkers. The PU-recovered group also had a higher proportion of participants engaging in regular exercise. Both groups had similar BMI, WC, blood pressure, and lipid profiles. However, the PU-developed group had a higher prevalence of diabetes, hypertension, and CKD.

### Incidence of CRC

**Fig. 3:**
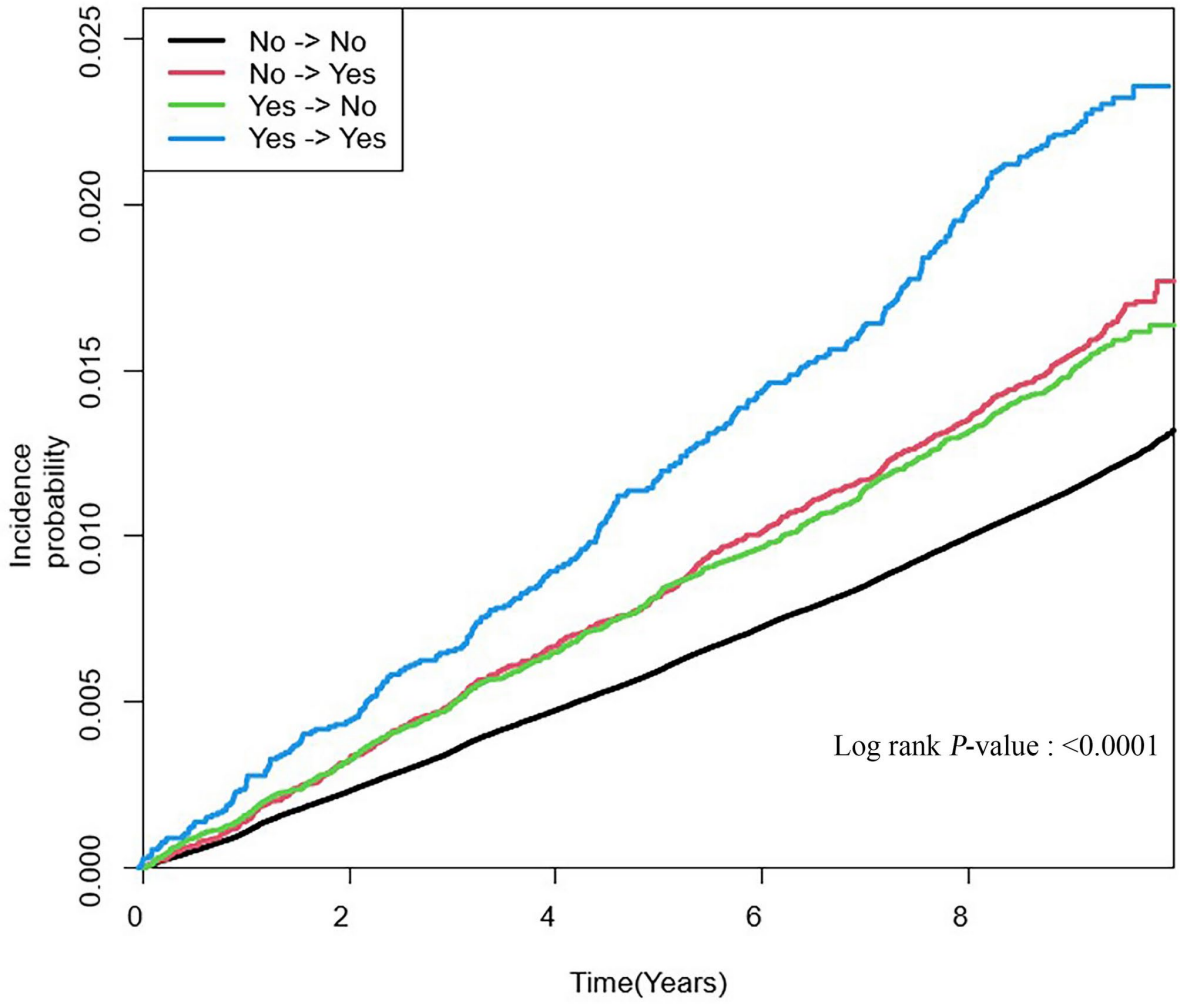
Kaplan-Meier curve for colorectal cancer (CRC) incidence by changes in proteinuria (PU) status. Kaplan-Meier analysis showing the cumulative incidence of colorectal cancer stratified by PU status groups (PU-free, PU-developed, PU-recovered, and PU-persistent). The PU-persistent group consistently exhibited the highest incidence of CRC over the follow-up period. Statistical significance was evaluated using the log-rank test (*p* < 0.001)

During the median follow-up period of 9.19 years, 36,846 (1.2%) individuals developed CRC. Table 2 shows the incidence rates and CRC risks based on changes in PU. The incidence rate of CRC per 1,000 person-years was highest in the PU-persistent group (2.46), followed by the PU-developed group (1.76), the PU-recovered group (1.68), and lowest in the PU-free group (1.29). As shown in the Kaplan-Meier curve in Fig. 3, the probability of developing CRC was consistently highest in the PU-persistent group and lowest in the PU-free group (log-rank *p* < 0.001).

**Table 2:** Incidence rates and colorectal cancer risk according to changes in proteinuria (PU) status

	*N*	Event	Duration	IR per 1,000	Model 1	Model 2	Model 3	Model 4	Model 5	Model 6
**Colorectal**										
Free	2,930,848	34,669	26949170.81	1.29	1 (ref.)	1 (ref.)	1 (ref.)	1 (ref.)	1 (ref.)	1 (ref.)
Developed	47,679	750	427040.17	1.76	1.37 (1.27, 1.47)	1.12 (1.04, 1.21)	1.11 (1.03, 1.19)	1.10 (1.02, 1.19)	1.07 (1.00, 1.15)	1.07 (1.00, 1.16)
Recovered	50,171	761	451810.89	1.68	1.31 (1.22, 1.41)	1.09 (1.02, 1.18)	1.08 (1.01, 1.16)	1.08 (1.00, 1.16)	1.05 (0.98, 1.13)	1.06 (0.98, 1.14)
Persistent	14,450	306	124384.75	2.46	1.92 (1.72, 2.15)	1.35 (1.21, 1.51)	1.33 (1.19, 1.49)	1.32 (1.18, 1.48)	1.26 (1.13, 1.41)	1.27 (1.13, 1.42)
*p*-value					< 0.0001	< 0.0001	< 0.0001	< 0.0001	< 0.0001	0.0008
*p* for trend					< 0.0001	< 0.0001	< 0.0001	< 0.0001	< 0.0001	< 0.0001
c-index					0.51	0.72	0.72	0.72	0.72	0.72
**Colon**										
Free	2,930,848	27,122	26949170.81	1.01	1 (ref.)	1 (ref.)	1 (ref.)	1 (ref.)	1 (ref.)	1 (ref.)
Developed	47,679	591	427040.17	1.38	1.38 (1.27, 1.49)	1.12 (1.03, 1.21)	1.10 (1.02, 1.20)	1.10 (1.01, 1.19)	1.07 (0.99, 1.16)	1.07 (0.99, 1.16)
Recovered	50,171	597	451810.89	1.32	1.31 (1.21, 1.43)	1.09 (1.00, 1.18)	1.07 (0.99, 1.17)	1.07 (0.99, 1.16)	1.05 (0.97, 1.14)	1.05 (0.97, 1.14)
Persistent	14,450	240	124384.75	1.93	1.92 (1.70, 2.19)	1.34 (1.18, 1.53)	1.33 (1.17, 1.51)	1.31 (1.16, 1.49)	1.26 (1.10, 1.43)	1.26 (1.11, 1.44)
*p*-value					< 0.0001	< 0.0001	< 0.0001	< 0.0001	0.001	0.0057
*p* for trend					< 0.0001	< 0.0001	< 0.0001	< 0.0001	0.0006	0.0005
c-index					0.51	0.73	0.73	0.73	0.73	0.73
**Rectum**										
Free	2,930,848	7547	26949170.81	0.28	1 (ref.)	1 (ref.)	1 (ref.)	1 (ref.)	1 (ref.)	1 (ref.)
Developed	47,679	159	427040.17	0.37	1.33 (1.14, 1.56)	1.13 (0.97, 1.32)	1.12 (0.96, 1.31)	1.12 (0.95, 1.31)	1.08 (0.92, 1.27)	1.09 (0.93, 1.27)
Recovered	50,171	164	451810.89	0.36	1.30 (1.11, 1.52)	1.13 (0.96, 1.31)	1.12 (0.96, 1.30)	1.11 (0.95, 1.30)	1.08 (0.93, 1.27)	1.09 (0.93, 1.27)
Persistent	14,450	66	124384.75	0.53	1.91 (1.50, 2.43)	1.37 (1.08, 1.75)	1.36 (1.07, 1.73)	1.35 (1.06, 1.72)	1.29 (1.01, 1.64)	1.30 (1.02, 1.67)
*p*-value					< 0.0001	0.0128	0.0206	0.0272	0.093	0.211
*p* for trend					< 0.0001	0.0018	0.0032	0.0044	0.0234	0.0184
c-index					0.51	0.68	0.69	0.69	0.69	0.69
**Right Colon**										
Free	2,930,848	4433	26949170.81	0.16	1 (ref.)	1 (ref.)	1 (ref.)	1 (ref.)	1 (ref.)	1 (ref.)
Developed	47,679	81	427040.17	0.19	1.16 (0.93, 1.44)	0.89 (0.72, 1.11)	0.88 (0.71, 1.10)	0.88 (0.70, 1.09)	0.85 (0.68, 1.06)	0.85 (0.68, 1.06)
Recovered	50,171	102	451810.89	0.23	1.38 (1.13, 1.67)	1.09 (0.89, 1.32)	1.07 (0.88, 1.30)	1.07 (0.88, 1.30)	1.04 (0.86, 1.27)	1.04 (0.86, 1.27)
Persistent	14,450	52	124384.75	0.42	2.56 (1.95, 3.36)	1.72 (1.31, 2.26)	1.67 (1.27, 2.20)	1.66 (1.26, 2.18)	1.57 (1.19, 2.08)	1.57 (1.19, 2.08)
*p*-value					< 0.0001	0.0008	0.0016	0.0019	0.0052	0.0076
*p* for trend					< 0.0001	0.0084	0.0188	0.0228	0.07	0.0713
c-index					0.51	0.78	0.78	0.78	0.78	0.78
**Left Colon**										
Free	2,930,848	6517	26949170.81	0.24	1 (ref.)	1 (ref.)	1 (ref.)	1 (ref.)	1 (ref.)	1 (ref.)
Developed	47,679	153	427040.17	0.36	1.48 (1.26, 1.74)	1.18 (1.00, 1.38)	1.15 (0.98, 1.35)	1.15 (0.97, 1.34)	1.10 (0.94, 1.30)	1.11 (0.94, 1.30)
Recovered	50,171	148	451810.89	0.33	1.35 (1.15, 1.59)	1.10 (0.94, 1.3)	1.08 (0.92, 1.28)	1.08 (0.91, 1.27)	1.05 (0.89, 1.23)	1.05 (0.89, 1.24)
Persistent	14,450	79	124384.75	0.64	2.63 (2.10, 3.28)	1.72 (1.37, 2.14)	1.69 (1.35, 2.10)	1.66 (1.33, 2.07)	1.56 (1.25, 1.95)	1.57 (1.26, 1.97)
*p*-value					< 0.0001	< 0.0001	< 0.0001	< 0.0001	0.0007	0.0045
*p* for trend					< 0.0001	< 0.0001	< 0.0001	< 0.0001	0.0014	0.0012
c-index					0.51	0.75	0.75	0.75	0.75	0.75

Model 1, unadjusted

Model 2, adjusted for age and sex

Model 3, additionally adjusted for smoking status, alcohol consumption, physical activity, and BMI

Model 4, additionally adjusted for the use of Renin-angiotensin system (RAS) inhibitors

Model 5, additionally adjusted for Diabetes, Hypertension, Dyslipidemia

Model 6, additionally adjusted for Chronic Kidney Disease

Compared with the PU-free group, the PU-persistent group had the highest risk of CRC (adjusted HR [aHR]: 1.32; 95% CI, 1.18–1.48; *p* < 0.0001), followed by the PU-developed group (aHR: 1.10; 95% CI, 1.02–1.19; *p* = 0.0076) and the PU-recovered group (aHR: 1.08; 95% CI, 1.00–1.16; *p* = 0.0419) (p for trend < 0.0001) (Model 4 of Table 2). Individuals in the PU-recovered group had a higher risk of CRC than subjects in the PU-free group; however, these individuals had a lower risk than subjects in the PU-persistent group. This trend persisted even when classified based on the site of the tumor except for right-sided colon cancer.

The risks of CRC based on the severity of PU were further analyzed. The incidence rate of CRC was highest in individuals with overt PU of + 2 to + 4 (2.13), showing a proportional relationship with the severity of PU. The risk of CRC also increased in proportion to the severity of PU, with the highest risk observed in the + 2 to + 4 PU group (aHR: 1.21; 95% CI, 1.09–1.34; *p* = 0.0003) (p for trend < 0.0001) (Model 4 of Table 3). A similar trend was found when analyzed based on tumor site except for right-sided colon cancer.

**Table 3:** Incidence rates and colorectal cancer risk according to the severity of proteinuria (PU)

Group	*N*	Event	Duration	IR per 1,000	Model 1		Model 2	Model 3	Model 4	Model 5	Model 6
**Colorectal**											
Neg	2,917,342	34,568	26820014.15	1.29	1 (ref.)		1 (ref.)	1 (ref.)	1 (ref.)	1 (ref.)	1 (ref.)
Trace	63,677	862	580967.55	1.48	1.15 (1.08, 1.23)		1.09 (1.02, 1.16)	1.08 (1.01, 1.15)	1.07 (1.00, 1.15)	1.06 (0.99, 1.14)	1.06 (0.99, 1.14)
Pos, +1	42,009	684	376637.76	1.82	1.41 (1.31, 1.52)		1.15 (1.07, 1.24)	1.14 (1.05, 1.23)	1.13 (1.05, 1.22)	1.10 (1.02, 1.19)	1.10 (1.02, 1.19)
Pos, + 2 ∼ 4	20,120	372	174787.16	2.13	1.66 (1.50, 1.83)		1.23 (1.11, 1.37)	1.22 (1.10, 1.35)	1.21 (1.09, 1.34)	1.16 (1.05, 1.29)	1.17 (1.05, 1.29)
*p*-value					< 0.0001		< 0.0001	< 0.0001	< 0.0001	0.0007	0.0076
*p* for trend					< 0.0001		< 0.0001	< 0.0001	< 0.0001	< 0.0001	< 0.0001
c-index					0.51		0.72	0.72	0.72	0.72	0.72
**Colon**											
Neg	2,917,342	27,047	26820014.15	1.01	1 (ref.)		1 (ref.)	1 (ref.)	1 (ref.)	1 (ref.)	1 (ref.)
Trace	63,677	672	580967.55	1.16	1.15 (1.06, 1.24)		1.08 (1.00, 1.17)	1.07 (0.99, 1.16)	1.07 (0.99, 1.16)	1.06 (0.98, 1.14)	1.06 (0.98, 1.14)
Pos, +1	42,009	536	376637.76	1.42	1.41 (1.30, 1.54)		1.14 (1.05, 1.24)	1.13 (1.04, 1.23)	1.12 (1.03, 1.22)	1.09 (1.00, 1.19)	1.09 (1.00, 1.19)
Pos, + 2 ∼ 4	20,120	295	174787.16	1.69	1.68 (1.50, 1.88)		1.24 (1.10, 1.39)	1.22 (1.09, 1.37)	1.21 (1.08, 1.36)	1.17 (1.04, 1.31)	1.17 (1.04, 1.31)
*p*-value					< 0.0001		< 0.0001	< 0.0001	0.0001	0.005	0.0257
*p* for trend					< 0.0001		< 0.0001	< 0.0001	< 0.0001	0.0005	0.0004
c-index					0.51		0.73	0.73	0.73	0.73	0.73
**Rectum**											
Neg	2,917,342	7521	26820014.15	0.28	1 (ref.)		1 (ref.)	1 (ref.)	1 (ref.)	1 (ref.)	1 (ref.)
Trace	63,677	190	580967.55	0.33	1.17 (1.01, 1.35)		1.10 (0.95, 1.27)	1.09 (0.95, 1.26)	1.09 (0.95, 1.26)	1.08 (0.93, 1.24)	1.08 (0.93, 1.25)
Pos, +1	42,009	148	376637.76	0.39	1.40 (1.19, 1.65)		1.18 (1.00, 1.39)	1.17 (0.99, 1.37)	1.16 (0.99, 1.37)	1.13 (0.96, 1.33)	1.13 (0.96, 1.33)
Pos, + 2 ∼ 4	20,120	77	174787.16	0.44	1.58 (1.26, 1.98)		1.22 (0.97, 1.53)	1.21 (0.96, 1.51)	1.20 (0.96, 1.50)	1.15 (0.92, 1.44)	1.16 (0.93, 1.45)
*p*-value					< 0.0001		0.0384	0.0604	0.073	0.2008	0.3896
*p* for trend					< 0.0001		0.004	0.007	0.0089	0.0409	0.0332
c-index					0.51		0.68	0.69	0.69	0.69	0.69
**Right Colon**											
Neg	2,917,342	4417	26820014.15	0.16	1 (ref.)		1 (ref.)	1 (ref.)	1 (ref.)	1 (ref.)	1 (ref.)
Trace	63,677	118	580967.55	0.20	1.23 (1.03, 1.48)		1.16 (0.96, 1.39)	1.15 (0.96, 1.38)	1.15 (0.96, 1.38)	1.13 (0.94, 1.36)	1.13 (0.94, 1.36)
Pos, +1	42,009	94	376637.76	0.25	1.52 (1.24, 1.86)		1.18 (0.96, 1.44)	1.16 (0.94, 1.42)	1.15 (0.94, 1.42)	1.12 (0.91, 1.37)	1.12 (0.91, 1.37)
Pos, + 2 ∼ 4	20,120	39	174787.16	0.22	1.36 (0.99, 1.87)		0.95 (0.70, 1.31)	0.93 (0.68, 1.28)	0.93 (0.68, 1.27)	0.89 (0.65, 1.22)	0.88 (0.64, 1.21)
*p*-value					< 0.0001		0.1761	0.2264	0.2389	0.3344	0.3659
*p* for trend					< 0.0001		0.1863	0.2759	0.3034	0.5604	0.5676
c-index					0.51		0.78	0.78	0.78	0.78	0.78
**Left Colon**											
Neg	2,917,342	6502	26820014.15	0.24	1 (ref.)		1 (ref.)	1 (ref.)	1 (ref.)	1 (ref.)	1 (ref.)
Trace	63,677	163	580967.55	0.28	1.16 (0.99, 1.35)		1.08 (0.92, 1.26)	1.06 (0.91, 1.24)	1.06 (0.91, 1.24)	1.04 (0.89, 1.21)	1.04 (0.89, 1.21)
Pos, +1	42,009	153	376637.76	0.41	1.68 (1.43, 1.97)		1.32 (1.12, 1.54)	1.29 (1.10, 1.51)	1.28 (1.09, 1.50)	1.24 (1.05, 1.45)	1.24 (1.05, 1.45)
Pos, + 2 ∼ 4	20,120	79	174787.16	0.45	1.86 (1.49, 2.33)		1.32 (1.05, 1.64)	1.29 (1.03, 1.61)	1.27 (1.02, 1.59)	1.21 (0.97, 1.51)	1.21 (0.97, 1.52)
*p*-value					< 0.0001		0.0006	0.0019	0.0033	0.0234	0.0916
*p* for trend					< 0.0001		< 0.0001	0.0002	0.0004	0.0041	0.0036
c-index					0.51		0.75	0.75	0.75	0.75	0.75

Model 1, unadjusted

Model 2, adjusted for age and sex

Model 3, additionally adjusted for smoking status, alcohol consumption, physical activity, and BMI

Model 4, additionally adjusted for the use of Renin-angiotensin system (RAS) inhibitors

Model 5, additionally adjusted for Diabetes, Hypertension, Dyslipidemia

Model 6, additionally adjusted for Chronic Kidney Disease

After additional adjustments for diabetes, hypertension, and dyslipidemia (Model 5), as well as chronic kidney disease (Model 6), the associations were slightly attenuated but remained significant for the PU-persistent group (aHR: 1.27; 95% CI, 1.13–1.42; *p* = 0.0008), became borderline significant for the PU-developed group (aHR: 1.07; 95% CI, 1.00–1.16; *p* = 0.0595), and were not significant for the PU-recovered group (aHR: 1.06; 95% CI, 0.98–1.14; *p* = 0.1543) (p for trend < 0.0001) (Model 6 of Table 2). Similar attenuation patterns were observed in analyses according to PU severity (Model 6, Table 3). In sensitivity analyses excluding participants diagnosed with diabetes mellitus and chronic kidney disease, the PU-persistent group remained significantly associated with increased CRC risk (aHR: 1.26; 95% CI, 1.03–1.53; *p* = 0.0247), demonstrating results consistent with the main analyses (Supplementary Table S2).

The C-index values for the Cox proportional hazards models ranged from 0.68 to 0.78, indicating moderate to good discriminatory power (Tables 2 and 3, and Supplementary Table S2). The proportional hazards assumption, as assessed by Schoenfeld residual tests, was satisfied for all covariates included in the final adjusted models (Supplementary Table S1). Calibration plots for the final adjusted model (Model 6) demonstrated good calibration, with predictions closely aligning to the diagonal reference line (Supplementary Figure S1). The optimism-corrected C-index was 0.719 (95% CI, 0.715–0.722), confirming robust internal validation and minimal overfitting.

### Subgroup analysis

Stratified analyses were performed based on age, sex, smoking status, drinking habits, diabetes, obesity, CKD, and intake of RAS inhibitors after adjusting for potential confounders; *p*-values for interactions are shown in Table 4 and Supplementary Table S3. Individuals with persistent PU faced higher risk of CRC across all subgroups. In addition, individuals in the PU-recovered group showed lower risk of CRC compared with subjects in the PU-developed group across all subgroups, with exceptions noted in females and participants with CKD. Notably, individuals < 65 years of age and males with persistent PU were at significantly higher risk of CRC (*p* for interaction = 0.0177 for age, and 0.0101 for sex, based on Model 6 in Supplementary Table S3).

**Table 4:** Subgroup analysis of colorectal cancer risk according to proteinuria (PU) status

Subgroup	Group	*N*	Event	Duration	IR per 1,000	Model 4	*p* for interation (Model 4)
Age < 65	No -> No	2,528,679	22,579	23459693.02	0.96	1 (ref.)	0.0168
	No -> Yes	37,391	442	343542.98	1.29	1.18 (1.08, 1.3)	
	Yes -> No	39,930	449	367410.75	1.22	1.12 (1.02, 1.23)	
	Yes -> Yes	10,544	180	95083.91	1.89	1.47 (1.26, 1.70)	
Age ≥ 65	No -> No	402,169	12,090	3489477.79	3.46	1 (ref.)	
	No -> Yes	10,288	308	83497.19	3.69	1.00 (0.90, 1.12)	
	Yes -> No	10,241	312	84400.14	3.70	1.02 (0.91, 1.14)	
	Yes -> Yes	3906	126	29300.84	4.30	1.15 (0.97, 1.38)	
Sex, male	No -> No	1,656,206	20,402	15163275.10	1.35	1 (ref.)	0.0044
	No -> Yes	27,042	491	239693.35	2.05	1.19 (1.09, 1.30)	
	Yes -> No	27,275	473	242747.48	1.95	1.14 (1.04, 1.25)	
	Yes -> Yes	9776	232	83143.18	2.79	1.41 (1.24, 1.60)	
Sex, female	No -> No	1,274,642	14,267	11785895.72	1.21	1 (ref.)	
	No -> Yes	20,637	259	187346.82	1.38	0.97 (0.86, 1.09)	
	Yes -> No	22,896	288	209063.41	1.38	0.99 (0.88, 1.11)	
	Yes -> Yes	4674	74	41241.58	1.79	1.11 (0.89, 1.40)	
Current Smoker (-)	No -> No	2,235,403	26,793	20577169.07	1.30	1 (ref.)	0.7728
	No -> Yes	36,572	567	328203.50	1.73	1.08 (1.00, 1.18)	
	Yes -> No	39,040	592	352257.42	1.68	1.08 (0.99, 1.17)	
	Yes -> Yes	10,843	234	93330.44	2.51	1.35 (1.18, 1.53)	
Current Smoker (+)	No -> No	695,445	7876	6372001.74	1.24	1 (ref.)	
	No -> Yes	11,107	183	98836.67	1.85	1.17 (1.01, 1.35)	
	Yes -> No	11,131	169	99553.47	1.70	1.08 (0.93, 1.26)	
	Yes -> Yes	3607	72	31054.31	2.32	1.24 (0.98, 1.57)	
Heavy Drinker (-)	No -> No	2,723,739	31,958	25052184.77	1.28	1 (ref.)	0.7441
	No -> Yes	43,538	670	390061.80	1.72	1.09 (1.01, 1.18)	
	Yes -> No	46,260	691	416666.13	1.66	1.07 (1.00, 1.16)	
	Yes -> Yes	13,186	275	113292.61	2.43	1.31 (1.16, 1.48)	
Heavy Drinker (+)	No -> No	207,109	2711	1896986.04	1.43	1 (ref.)	
	No -> Yes	4141	80	36978.37	2.16	1.23 (0.98, 1.53)	
	Yes -> No	3911	70	35144.76	1.99	1.12 (0.89, 1.42)	
	Yes -> Yes	1264	31	11092.14	2.79	1.42 (0.99, 2.02)	
DM (-)	No -> No	2,650,203	28,610	24446166.20	1.17	1 (ref.)	0.8297
	No -> Yes	35,845	462	326078.84	1.42	1.05 (0.96, 1.15)	
	Yes -> No	38,787	493	353753.50	1.39	1.04 (0.95, 1.14)	
	Yes -> Yes	8361	143	74255.90	1.93	1.26 (1.07, 1.49)	
DM (+)	No -> No	280,645	6059	2503004.62	2.42	1 (ref.)	
	No -> Yes	11,834	288	100961.33	2.85	1.12 (0.99, 1.26)	
	Yes -> No	11,384	268	98057.39	2.73	1.08 (0.96, 1.22)	
	Yes -> Yes	6089	163	50128.85	3.25	1.26 (1.08, 1.48)	
Obesity (-)	No -> No	1,945,438	21,922	17881735.73	1.23	1 (ref.)	0.9978
	No -> Yes	28,084	416	250331.24	1.66	1.1 (1.00, 1.21)	
	Yes -> No	29,518	426	264794.36	1.61	1.08 (0.99, 1.19)	
	Yes -> Yes	7244	150	61028.65	2.46	1.32 (1.13, 1.55)	
Obesity (+)	No -> No	985,410	12,747	9067435.08	1.41	1 (ref.)	
	No -> Yes	19,595	334	176708.93	1.89	1.10 (0.99, 1.23)	
	Yes -> No	20,653	335	187016.53	1.79	1.07 (0.96, 1.19)	
	Yes -> Yes	7206	156	63356.10	2.46	1.32 (1.13, 1.54)	
CKD (-)	No -> No	2,831,452	32,480	26078222.86	1.25	1 (ref.)	0.1683
	No -> Yes	42,523	647	384426.54	1.68	1.14 (1.05, 1.23)	
	Yes -> No	45,697	668	414623.56	1.61	1.1 (1.02, 1.19)	
	Yes -> Yes	10,310	202	91297.49	2.21	1.31 (1.14, 1.51)	
CKD (+)	No -> No	99,396	2189	870947.95	2.51	1 (ref.)	
	No -> Yes	5156	103	42613.63	2.42	0.93 (0.77, 1.14)	
	Yes -> No	4474	93	37187.33	2.50	0.94 (0.77, 1.16)	
	Yes -> Yes	4140	104	33087.26	3.14	1.35 (1.11, 1.64)	
RAS Inhibitor (-)	No -> No	2,419,118	24,505	22346341.41	1.10	1 (ref.)	0.8552
	No -> Yes	32,165	403	292969.88	1.38	1.10 (1.00, 1.21)	
	Yes -> No	32,740	403	299397.48	1.35	1.11 (1.01, 1.23)	
	Yes -> Yes	6187	104	55033.07	1.89	1.30 (1.08, 1.58)	
RAS Inhibitor (+)	No -> No	511,730	10,164	4602829.41	2.21	1 (ref.)	
	No -> Yes	15,514	347	134070.29	2.59	1.11 (0.99, 1.23)	
	Yes -> No	17,431	358	152413.41	2.35	1.04 (0.94, 1.16)	
	Yes -> Yes	8263	202	69351.69	2.91	1.33 (1.16, 1.53)	

## Discussion

In this large-scale, population-based cohort study, the significant effects of PU changes on the incidence of CRC were highlighted. PU was independently associated with an increased risk of CRC in the general population. After adjusting comprehensively for confounding factors, including age, sex, lifestyle behaviors, medication use, diabetes, hypertension, dyslipidemia, and chronic kidney disease, persistent PU remained significantly associated with increased CRC risk. Notably, even individuals who recovered from PU still faced a higher risk of CRC than subjects free of PU; however, their risk was lower than that of individuals with persistent PU. Furthermore, the risk of CRC progressively increased with greater severity of PU, demonstrating a dose-dependent relationship. Although adjustments for diabetes, hypertension, dyslipidemia, and chronic kidney disease could potentially lead to concerns about over-adjustment, our sensitivity analyses excluding participants with diabetes mellitus and chronic kidney disease produced consistent findings, supporting the validity of these adjusted associations. These findings emphasize the clinical relevance of proteinuria as a potentially modifiable risk factor for colorectal cancer, highlighting the importance of regular monitoring and early management of PU in clinical practice.

Several epidemiological studies have investigated the associations between proteinuria (PU), albuminuria, and colorectal cancer (CRC), with varying methodologies and outcomes. Kim et al. (2013) [17] found that proteinuria significantly increased cancer-specific mortality among patients with early-stage colorectal cancer (hazard ratio: 1.67; 95% CI, 1.15–2.42). Matsuoka et al. (2022) [22] reported an increased risk of incident colorectal cancer among individuals with trace or overt proteinuria in a large Japanese population-based study. Similarly, Ahn et al. (2020) [23], using Korean National Health Insurance data, showed that even dipstick-positive proteinuria was significantly associated with a higher risk of colorectal cancer incidence. Despite these findings, previous studies largely relied on cross-sectional assessments of proteinuria, limiting the ability to explore how dynamic changes in proteinuria status affect CRC risk. As PU can be reversible, our study expands on this research by presenting findings from longitudinal proteinuria monitoring that show even temporary changes in PU status, whether in development or resolution, are associated with significant risk of CRC. The study indicates that modifying PU status, probably through pharmacological interventions, lifestyle changes, or management of underlying conditions, could potentially reduce the risk of CRC. While earlier research considered PU as a prognostic factor, our study shifts the focus to PU as a potential early indicator for cancer development. This emphasizes the importance of early detection and management PU in individuals with chronic conditions such as diabetes and hypertension, which may serve as a marker for cancer prevention strategies.

The exact mechanisms linking PU to CRC remain unclear but are likely multifactorial. It’s been widely reported how reduced kidney function affects the incidence of various cancers such as urothelial cancers, hematologic cancers, and gastrointestinal cancers, as well as increasing cancer mortality [24–26]. Albuminuria was reported to be independently associated with cancer risk, specifically 9–66% increase in risk of incident cancer. This association has been consistently observed across different populations, including East Asian cohorts (Taiwanese and Korean) and European populations (predominantly Caucasian), regardless of whether proteinuria is identified semi-quantitatively on dipstick testing or quantitatively by urinary albumin-to-creatinine ratio, demonstrating a dose–response relationship [24, 26–28]. For instance, Mok et al. [26] additionally reported high PU was also associated with cancer risk, showing a dose-response relationship (HRs of 1.24 [95% CI, 1.13–1.35], 1.38 [95% CI, 1.17–1.63], and 1.66 [95% CI, 1.30–2.12] for 1+, 2+, and ≥ 3 + vs. undetectable/trace) which is consistent with our study. PU is generally considered a marker of endothelial dysfunction and systemic inflammation, both of which are implicated in carcinogenesis. PU serves as an early indicator of systemic vascular endothelial dysfunction and glomerular endothelia damage [29, 30]. The inflammatory microenvironment created by kidney dysfunction contributes to chronic inflammation and oxidative stress, both of which may facilitate cancer development [31–33]. Inflammatory processes can promote cancer initiation and progression by inducing DNA damage, inhibiting apoptosis, and creating a pro-tumorigenic environment. Specifically, chronic kidney dysfunction and persistent proteinuria lead to a pro-inflammatory environment characterized by elevated cytokines (e.g., IL-6, TNF-α), increased expression of growth factors such as vascular endothelial growth factor (VEGF), and activation of critical signaling pathways, including NF-κB [34, 35]. Elevated cytokines such as IL-6 and TNF-α promote colorectal tumor initiation and progression via inflammatory and proliferative mechanisms [36]. VEGF is closely associated with tumor angiogenesis, facilitating tumor growth and metastasis in colorectal cancer [37]. NF-κB activation, a key inflammatory signaling pathway, promotes carcinogenesis by enhancing cell proliferation, inhibiting apoptosis, and sustaining tumor-associated inflammation [38]. Furthermore, the deposition of tumor-derived antigens and immune complexes in glomerular capillaries may activate inflammatory cascades and induce glomerular damage [39, 40]. In addition, PU is often associated with metabolic syndrome, diabetes, and CKD, all of which are recognized risk factors for malignancies, including CRC [41–45]. The increased risk observed in the present study may be explained by the higher prevalence of these comorbidities among individuals with PU.

Notably, although the overall trend showed a significant association between PU and CRC, our findings demonstrated a weaker association between proteinuria (PU) and right-sided colon cancer compared to left-sided colon cancer or rectal cancer. This observation aligns with accumulating evidence that colorectal cancers arising in different anatomical subsites exhibit distinct clinical presentations, molecular characteristics, and underlying risk factors. Specifically, right-sided colon cancers commonly show high levels of microsatellite instability (MSI), CpG island methylator phenotype (CIMP), and frequent BRAF mutations, whereas left-sided colon cancers predominantly feature chromosomal instability, alterations in APC and KRAS genes, and different methylation patterns [46, 47]. Additionally, differences in colorectal cancer risk factors according to anatomical location have been reported by multinational cohort studies, suggesting distinct etiological pathways along the colon [48]. For example, right-sided colon cancers have been associated with dietary factors, genetic predispositions, and immune-mediated inflammation, whereas left-sided colon cancers have stronger correlations with lifestyle-related risk factors such as alcohol intake, smoking, and obesity. Furthermore, environmental variations within the colon, such as differential exposure to microbial communities, bile acids, fermentation products, and regional differences in luminal pH, may also modulate carcinogenic processes and contribute to site-specific tumorigenesis [49]. Future studies focusing on these anatomical subsites, employing molecular profiling and detailed assessment of environmental and inflammatory markers, could further elucidate the mechanisms underlying these differential associations.

Although prior studies have indicated an association between proteinuria (PU) and colorectal cancer (CRC), this study is, to our knowledge, the first to specifically evaluate the impact of dynamic changes in proteinuria status on colorectal cancer incidence using longitudinal data from a nationwide cohort. Unlike previous studies, which typically assessed PU at a single time point, our research uniquely investigates changes in PU and their corresponding risk profiles for CRC development. However, our study has several limitations. First, due to limited data, certain potential confounding factors, such as family history of CRC, genetic predispositions, inflammatory bowel disease, dietary exposure information, and colonoscopy history, could not be investigated, possibly introducing further residual confounding. Moreover, information on the stage or histopathological type of CRC was not included in the analysis due to a lack of available data. As our data were derived from the nationwide National Health Insurance Service database, detailed personal information such information was inherently unavailable, reflecting a common limitation of large-scale administrative databases. In addition, residual confounding due to undiagnosed or unmeasured metabolic or inflammatory conditions might persist, despite comprehensive adjustments and sensitivity analyses. Future studies should consider including these additional variables to further clarify the relationship observed. Second, as all baseline variables, including demographic, clinical, and behavioral factors, were measured based on the second screening (2012), any changes in behavioral status, such as smoking, drinking, or physical activity, that occurred between the first (2010) and second screening were not captured in our analysis. This limitation could potentially introduce misclassification bias regarding these behaviors and consequently affect the associations observed. Additionally, internal validation methods such as bootstrapping or cross-validation were not conducted in this study due to the administrative nature and large-scale structure of the dataset. Third, although we emphasized the dynamic nature of proteinuria (PU) changes, our analysis was limited to capturing only a single interval between two consecutive screenings (2010 and 2012). Therefore, we could not assess further fluctuations or subsequent changes in PU status, which might have occurred throughout the follow-up period. Moreover, due to the inherent limitations of this large-scale administrative cohort, we were unable to precisely evaluate the exact timing or duration from initial proteinuria onset to colorectal cancer diagnosis. Future research employing multiple longitudinal assessments and more frequent or continuous monitoring of PU and repeated assessments of behavioral factors could better elucidate the clinical significance of dynamic PU changes. Fourth, while we conducted a semiquantitative assessment using the urine dipstick test, current guidelines recommend quantitative assessment of proteinuria through methods such as the random urine protein-to-creatinine ratio, urine albumin-to-creatinine ratio (UACR), or 24-hour urine collection [50, 51]. Dipstick urinalysis provides semiquantitative results and is inherently subject to variability, including potential false-positive or false-negative readings. Such variability may lead to misclassification bias, potentially resulting in an underestimation or overestimation of the true association between proteinuria and colorectal cancer risk. Quantitative methods, such as UACR or 24-hour urine protein measurement, provide more precise and reproducible assessments. For instance, Jørgensen et al. demonstrated a clear dose-response relationship between UACR-measured proteinuria and increased cancer incidence, highlighting the greater precision offered by quantitative methods compared to dipstick testing [28]. Although direct comparison of our findings with quantitative studies is limited due to methodological differences, our results are broadly consistent in indicating elevated colorectal cancer risk with increasing proteinuria severity. Although dipstick urinalysis remains highly practical and efficient for large-scale screening purposes [52], further studies employing more precise proteinuria measurements could strengthen the evidence regarding the association between proteinuria and colorectal cancer risk. Fifth, this study was conducted using data exclusively from an ethnic Korean population. Therefore, the generalizability of our findings to other ethnic groups may be limited. Ethnic and geographic variations could influence genetic predispositions, prevalence and etiology of proteinuria, lifestyle factors, healthcare access, and cancer screening behaviors. Thus, caution should be exercised when generalizing our results beyond this context. Future studies involving diverse populations are needed to validate our findings and to explore potential interactions with demographic and geographic characteristics. Lastly, as this study is observational, it does not fully establish a causal relationship between changes in proteinuria and colorectal cancer incidence. Future studies utilizing different methodologies, such as randomized controlled trials (RCTs) to evaluate interventions targeting proteinuria reduction, or Mendelian randomization (MR) analyses leveraging genetic variants associated with proteinuria, may provide stronger evidence regarding causality. Despite the limitations outlined above, this study’s strength lies in its large-scale, nationwide cohort design and the use of standardized health data from a highly representative population, allowing for robust analysis of PU status changes over time and their association with colorectal cancer risk. By utilizing longitudinal data, we were able to account for dynamic changes in PU, offering unique insights into PU as a potentially modifiable cancer risk factor.

## Conclusions

Our study highlights the significant association between changes in PU status and CRC risk. Persistent proteinuria poses the greatest risk, and even transient changes in PU status were associated with elevated cancer incidence. These findings indicate that regular monitoring of proteinuria and interventions aimed at controlling PU may contribute to colorectal cancer prevention, especially in high-risk individuals. However, further research including interventional studies such as randomized controlled trials, is warranted to confirm the causality and effectiveness of PU management strategies in reducing CRC risk across diverse populations.

## Electronic supplementary material

Below is the link to the electronic supplementary material.

Supplementary Material 1

## Data Availability

The data that support the findings of this study are available from the Korea National Health Insurance Service (NHIS) database (https://nhiss.nhis.or.kr), but restrictions apply to the availability of these data, which were used under license for the current study. Therefore, the data are not publicly available. Data are, however, available from the authors upon reasonable request and with permission from the NHIS.
