# Supplementary material for: Association between proteinuria changes and colorectal cancer incidence: evidence from a nationwide cohort study
Source: BMC Gastroenterol. 2025 May 21;25:392. doi: 10.1186/s12876-025-03935-7 (PMC12093895; doi:10.1186/s12876-025-03935-7)
Supplement: Supplementary file 1 — Supplementary Material 1 [file 12876_2025_3935_MOESM1_ESM.docx]

Supplementary Table S1. Test of proportional hazards assumption (*p*-values) for each Cox regression model and cancer outcome

| Outcome | Model 1 | Model 2 | Model 3 | Model 4 | Model 5 | Model 6 |
| --- | --- | --- | --- | --- | --- | --- |
| Colorectal | 0.00 | 0.90 | 0.86 | 0.84 | 0.88 | 0.78 |
| Colon | 0.46 | 0.64 | 0.66 | 0.69 | 0.67 | 0.76 |
| Rectum | 0.02 | 0.25 | 0.24 | 0.25 | 0.27 | 0.25 |
| Proximal Colon | 0.26 | 0.58 | 0.59 | 0.67 | 0.62 | 0.63 |
| Distal Colon | 0.41 | 0.11 | 0.11 | 0.11 | 0.10 | 0.12 |
| Unspecified | 0.39 | 0.94 | 0.92 | 0.81 | 0.85 | 0.77 |

Supplementary Table S2. Sensitivity analysis of colorectal cancer risk according to changes in proteinuria (PU) status after excluding participants with diabetes mellitus (DM) and chronic kidney disease (CKD) (n=2,649,576)

| Group | N | Event | Duration | IR per  1,000 | Model 1 | Model 2 | Model 3 | Model 4 | Model 5 |
| --- | --- | --- | --- | --- | --- | --- | --- | --- | --- |
| **Colorectal** |  |  |  |  |  |  |  |  |  |
| Free | 2574318 | 27111 | 23771878.52 | 1.14 | 1 (ref.) | 1 (ref.) | 1 (ref.) | 1 (ref.) | 1 (ref.) |
| Developed | 32807 | 413 | 300062.81 | 1.38 | 1.21 (1.09, 1.33) | 1.09 (0.99, 1.21) | 1.08 (0.98, 1.19) | 1.08 (0.98, 1.19) | 1.08 (0.98, 1.19) |
| Recovered | 36172 | 451 | 331216.36 | 1.36 | 1.20 (1.09, 1.31) | 1.08 (0.98, 1.18) | 1.07 (0.97, 1.17) | 1.07 (0.97, 1.17) | 1.07 (0.97, 1.17) |
| Persistent | 6279 | 98 | 56687.31 | 1.73 | 1.52 (1.25, 1.86) | 1.28 (1.05, 1.56) | 1.25 (1.03, 1.53) | 1.26 (1.03, 1.53) | 1.26 (1.03, 1.53) |
| c-index |  |  |  |  | 0.50 | 0.71 | 0.71 | 0.71 | 0.71 |
| **Colon** |  |  |  |  |  |  |  |  |  |
| Free | 2574318 | 21066 | 23771878.52 | 0.89 | 1 (ref.) | 1 (ref.) | 1 (ref.) | 1 (ref.) | 1 (ref.) |
| Developed | 32807 | 328 | 300062.81 | 1.09 | 1.23 (1.11, 1.38) | 1.11 (0.99, 1.24) | 1.10 (0.98, 1.22) | 1.10 (0.98, 1.23) | 1.10 (0.98, 1.22) |
| Recovered | 36172 | 352 | 331216.36 | 109.21 | 1.2 (1.08, 1.33) | 1.07 (0.96, 1.19) | 1.06 (0.96, 1.18) | 1.06 (0.96, 1.18) | 1.06 (0.96, 1.18) |
| Persistent | 6279 | 79 | 56687.31 | 110.77 | 1.57 (1.26, 1.96) | 1.32 (1.06, 1.65) | 1.30 (1.04, 1.62) | 1.30 (1.04, 1.63) | 1.30 (1.05, 1.63) |
| c-index |  |  |  |  | 0.50 | 0.73 | 0.73 | 0.73 | 0.73 |
| **Rectum** |  |  |  |  |  |  |  |  |  |
| Free | 2574318 | 6045 | 23771878.52 | 0.25 | 1 (ref.) | 1 (ref.) | 1 (ref.) | 1 (ref.) | 1 (ref.) |
| Developed | 32807 | 85 | 300062.81 | 0.28 | 1.11 (0.9, 1.38) | 1.04 (0.84, 1.29) | 1.03 (0.83, 1.27) | 1.03 (0.83, 1.27) | 1.02 (0.83, 1.27) |
| Recovered | 36172 | 99 | 331216.36 | 0.30 | 1.18 (0.96, 1.44) | 1.10 (0.90, 1.34) | 1.09 (0.90, 1.33) | 1.09 (0.90, 1.33) | 1.09 (0.89, 1.33) |
| Persistent | 6279 | 19 | 56687.31 | 0.34 | 1.32 (0.84, 2.07) | 1.11 (0.71, 1.75) | 1.10 (0.70, 1.72) | 1.09 (0.70, 1.72) | 1.09 (0.69, 1.71) |
| c-index |  |  |  |  | 0.50 | 0.68 | 0.68 | 0.68 | 0.68 |
| **Right Colon** |  |  |  |  |  |  |  |  |  |
| Free | 2574318 | 3333 | 23771878.52 | 0.14 | 1 (ref.) | 1 (ref.) | 1 (ref.) | 1 (ref.) | 1 (ref.) |
| Developed | 32807 | 48 | 300062.81 | 0.16 | 1.14 (0.86, 1.52) | 0.99 (0.74, 1.31) | 0.98 (0.74, 1.30) | 0.98 (0.74, 1.31) | 0.98 (0.74, 1.30) |
| Recovered | 36172 | 52 | 331216.36 | 0.16 | 1.12 (0.85, 1.47) | 0.96 (0.73, 1.27) | 0.95 (0.73, 1.25) | 0.96 (0.73, 1.26) | 0.96 (0.73, 1.26) |
| Persistent | 6279 | 16 | 56687.31 | 0.28 | 2.02 (1.24, 3.30) | 1.65 (1.01, 2.69) | 1.61 (0.99, 2.64) | 1.62 (0.99, 2.66) | 1.62 (0.99, 2.65) |
| c-index |  |  |  |  | 0.50 | 0.78 | 0.78 | 0.78 | 0.78 |
| **Left** **Colon** |  |  |  |  |  |  |  |  |  |
| Free | 2574318 | 4981 | 23771878.52 | 0.21 | 1 (ref.) | 1 (ref.) | 1 (ref.) | 1 (ref.) | 1 (ref.) |
| Developed | 32807 | 70 | 300062.81 | 0.23 | 1.11 (0.88, 1.41) | 0.99 (0.78, 1.25) | 0.97 (0.77, 1.23) | 0.97 (0.77, 1.23) | 0.97 (0.77, 1.23) |
| Recovered | 36172 | 82 | 331216.36 | 0.25 | 1.18 (0.95, 1.47) | 1.05 (0.85, 1.31) | 1.04 (0.84, 1.29) | 1.04 (0.84, 1.29) | 1.04 (0.83, 1.29) |
| Persistent | 6279 | 26 | 56687.31 | 0.46 | 2.19 (1.49, 3.22) | 1.75 (1.19, 2.57) | 1.71 (1.16, 2.51) | 1.70 (1.16, 2.50) | 1.70 (1.16, 2.50) |
| c-index |  |  |  |  | 0.50 | 0.74 | 0.75 | 0.75 | 0.75 |

Supplementary Table S3. Subgroup analysis of colorectal cancer risk according to proteinuria (PU) status.

| Subgroup | Group | N | Event | Duration | IR per 1,000 | Model 5 | *p* for interation (Model 5) | Model 6 | *p* for interation (Model 6) |
| --- | --- | --- | --- | --- | --- | --- | --- | --- | --- |
| Age < 65 | No -> No | 2528679 | 22579 | 23459693.02 | 0.96 | 1 (ref.) | 0.0164 | 1 (ref.) | 0.0177 |
|  | No -> Yes | 37391 | 442 | 343542.98 | 1.29 | 1.15 (1.05, 1.26) |  | 1.15 (1.05, 1.27) |  |
|  | Yes -> No | 39930 | 449 | 367410.75 | 1.22 | 1.10 (1.00, 1.20) |  | 1.10 (1.00, 1.21) |  |
|  | Yes -> Yes | 10544 | 180 | 95083.91 | 1.89 | 1.40 (1.21, 1.62) |  | 1.40 (1.21, 1.63) |  |
| Age ≥ 65 | No -> No | 402169 | 12090 | 3489477.79 | 3.46 | 1 (ref.) |  | 1 (ref.) |  |
|  | No -> Yes | 10288 | 308 | 83497.19 | 3.69 | 0.98 (0.87, 1.09) |  | 0.98 (0.87, 1.09) |  |
|  | Yes -> No | 10241 | 312 | 84400.14 | 3.70 | 1.00 (0.89, 1.12) |  | 1 (0.89, 1.12) |  |
|  | Yes -> Yes | 3906 | 126 | 29300.84 | 4.30 | 1.10 (0.92, 1.31) |  | 1.11 (0.93, 1.32) |  |
| Sex, male | No -> No | 1656206 | 20402 | 15163275.10 | 1.35 | 1 (ref.) | 0.0099 | 1 (ref.) | 0.0101 |
|  | No -> Yes | 27042 | 491 | 239693.35 | 2.05 | 1.15 (1.05, 1.26) |  | 1.15 (1.05, 1.26) |  |
|  | Yes -> No | 27275 | 473 | 242747.48 | 1.95 | 1.11 (1.01, 1.22) |  | 1.11 (1.01, 1.22) |  |
|  | Yes -> Yes | 9776 | 232 | 83143.18 | 2.79 | 1.34 (1.18, 1.52) |  | 1.35 (1.18, 1.53) |  |
| Sex, female | No -> No | 1274642 | 14267 | 11785895.72 | 1.21 | 1 (ref.) |  | 1 (ref.) |  |
|  | No -> Yes | 20637 | 259 | 187346.82 | 1.38 | 0.95 (0.84, 1.07) |  | 0.95 (0.84, 1.07) |  |
|  | Yes -> No | 22896 | 288 | 209063.41 | 1.38 | 0.98 (0.87, 1.10) |  | 0.98 (0.87, 1.10) |  |
|  | Yes -> Yes | 4674 | 74 | 41241.58 | 1.79 | 1.08 (0.86, 1.35) |  | 1.08 (0.86, 1.36) |  |
| Current Smoker (-) | No -> No | 2235403 | 26793 | 20577169.07 | 1.30 | 1 (ref.) | 0.79 | 1 (ref.) | 0.7885 |
|  | No -> Yes | 36572 | 567 | 328203.50 | 1.73 | 1.05 (0.97, 1.15) |  | 1.06 (0.97, 1.15) |  |
|  | Yes -> No | 39040 | 592 | 352257.42 | 1.68 | 1.06 (0.97, 1.15) |  | 1.06 (0.97, 1.15) |  |
|  | Yes -> Yes | 10843 | 234 | 93330.44 | 2.51 | 1.29 (1.13, 1.47) |  | 1.30 (1.14, 1.48) |  |
| Current Smoker (+) | No -> No | 695445 | 7876 | 6372001.74 | 1.24 | 1 (ref.) |  | 1 (ref.) |  |
|  | No -> Yes | 11107 | 183 | 98836.67 | 1.85 | 1.13 (0.97, 1.30) |  | 1.13 (0.97, 1.31) |  |
|  | Yes -> No | 11131 | 169 | 99553.47 | 1.70 | 1.05 (0.90, 1.23) |  | 1.05 (0.91, 1.23) |  |
|  | Yes -> Yes | 3607 | 72 | 31054.31 | 2.32 | 1.18 (0.93, 1.49) |  | 1.18 (0.94, 1.49) |  |
| Heavy Drinker (-) | No -> No | 2723739 | 31958 | 25052184.77 | 1.28 | 1 (ref.) | 0.7888 | 1 (ref.) | 0.8 |
|  | No -> Yes | 43538 | 670 | 390061.80 | 1.72 | 1.06 (0.98, 1.15) |  | 1.06 (0.98, 1.15) |  |
|  | Yes -> No | 46260 | 691 | 416666.13 | 1.66 | 1.05 (0.97, 1.13) |  | 1.05 (0.98, 1.14) |  |
|  | Yes -> Yes | 13186 | 275 | 113292.61 | 2.43 | 1.25 (1.11, 1.41) |  | 1.26 (1.12, 1.42) |  |
| Heavy Drinker (+) | No -> No | 207109 | 2711 | 1896986.04 | 1.43 | 1 (ref.) |  | 1 (ref.) |  |
|  | No -> Yes | 4141 | 80 | 36978.37 | 2.16 | 1.18 (0.95, 1.48) |  | 1.19 (0.95, 1.48) |  |
|  | Yes -> No | 3911 | 70 | 35144.76 | 1.99 | 1.09 (8.6, 1.38) |  | 1.09 (0.86, 1.38) |  |
|  | Yes -> Yes | 1264 | 31 | 11092.14 | 2.79 | 1.35 (0.95, 1.92) |  | 1.35 (0.95, 1.93) |  |
| DM (-) | No -> No | 2650203 | 28610 | 24446166.20 | 1.17 | 1 (ref.) | 0.8159 | 1 (ref.) | 0.8078 |
|  | No -> Yes | 35845 | 462 | 326078.84 | 1.42 | 1.05 (0.95, 1.15) |  | 1.05 (0.96, 1.15) |  |
|  | Yes -> No | 38787 | 493 | 353753.50 | 1.39 | 1.04 (0.95, 1.14) |  | 1.04 (0.95, 1.14) |  |
|  | Yes -> Yes | 8361 | 143 | 74255.90 | 1.93 | 1.26 (1.07, 1.49) |  | 1.27 (1.08, 1.50) |  |
| DM (+) | No -> No | 280645 | 6059 | 2503004.62 | 2.42 | 1 (ref.) |  | 1 (ref.) |  |
|  | No -> Yes | 11834 | 288 | 100961.33 | 2.85 | 1.12 (0.99, 1.26) |  | 1.12 (0.99, 1.26) |  |
|  | Yes -> No | 11384 | 268 | 98057.39 | 2.73 | 1.08 (0.96, 1.23) |  | 1.09 (0.96, 1.23) |  |
|  | Yes -> Yes | 6089 | 163 | 50128.85 | 3.25 | 1.27 (1.08, 1.48) |  | 1.28 (1.09, 1.49) |  |
| Obesity (-) | No -> No | 1945438 | 21922 | 17881735.73 | 1.23 | 1 (ref.) | 0.997 | 1 (ref.) | 0.9971 |
|  | No -> Yes | 28084 | 416 | 250331.24 | 1.66 | 1.07 (0.97, 1.18) |  | 1.07 (0.97, 1.18) |  |
|  | Yes -> No | 29518 | 426 | 264794.36 | 1.61 | 1.06 (0.96, 1.17) |  | 1.06 (0.97, 1.17) |  |
|  | Yes -> Yes | 7244 | 150 | 61028.65 | 2.46 | 1.26 (1.07, 1.48) |  | 1.27 (1.08, 1.49) |  |
| Obesity (+) | No -> No | 985410 | 12747 | 9067435.08 | 1.41 | 1 (ref.) |  | 1 (ref.) |  |
|  | No -> Yes | 19595 | 334 | 176708.93 | 1.89 | 1.07 (0.96, 1.20) |  | 1.07 (0.96, 1.20) |  |
|  | Yes -> No | 20653 | 335 | 187016.53 | 1.79 | 1.04 (0.94, 1.17) |  | 1.05 (0.94, 1.17) |  |
|  | Yes -> Yes | 7206 | 156 | 63356.10 | 2.46 | 1.26 (1.08, 1.48) |  | 1.27 (1.08, 1.49) |  |
| CKD (-) | No -> No | 2831452 | 32480 | 26078222.86 | 1.25 | 1 (ref.) | 0.1589 | 1 (ref.) | 0.1589 |
|  | No -> Yes | 42523 | 647 | 384426.54 | 1.68 | 1.10 (1.02, 1.19) |  | 1.10 (1.02, 1.19) |  |
|  | Yes -> No | 45697 | 668 | 414623.56 | 1.61 | 1.08 (1.00, 1.16) |  | 1.08 (1.00, 1.16) |  |
|  | Yes -> Yes | 10310 | 202 | 91297.49 | 2.21 | 1.25 (1.09, 1.44) |  | 1.25 (1.09, 1.44) |  |
| CKD (+) | No -> No | 99396 | 2189 | 870947.95 | 2.51 | 1 (ref.) |  | 1 (ref.) |  |
|  | No -> Yes | 5156 | 103 | 42613.63 | 2.42 | 0.91 (0.74, 1.11) |  | 0.91 (0.74, 1.11) |  |
|  | Yes -> No | 4474 | 93 | 37187.33 | 2.50 | 0.92 (0.75, 1.13) |  | 0.92 (0.75, 1.13) |  |
|  | Yes -> Yes | 4140 | 104 | 33087.26 | 3.14 | 1.30 (1.07, 1.58) |  | 1.30 (1.07, 1.58) |  |
| RAS Inhibitor (-) | No -> No | 2419118 | 24505 | 22346341.41 | 1.10 | 1 (ref.) | 0.8127 | 1 (ref.) | 0.8192 |
|  | No -> Yes | 32165 | 403 | 292969.88 | 1.38 | 1.07 (0.97, 1.18) |  | 1.07 (0.97, 1.18) |  |
|  | Yes -> No | 32740 | 403 | 299397.48 | 1.35 | 1.09 (0.99, 1.20) |  | 1.09 (0.99, 1.20) |  |
|  | Yes -> Yes | 6187 | 104 | 55033.07 | 1.89 | 1.24 (1.02, 1.51) |  | 1.25 (1.03, 1.51) |  |
| RAS Inhibitor (+) | No -> No | 511730 | 10164 | 4602829.41 | 2.21 | 1 (ref.) |  | 1 (ref.) |  |
|  | No -> Yes | 15514 | 347 | 134070.29 | 2.59 | 1.07 (0.96, 1.20) |  | 1.08 (0.97, 1.2) |  |
|  | Yes -> No | 17431 | 358 | 152413.41 | 2.35 | 1.02 (0.92, 1.13) |  | 1.02 (0.92, 1.13) |  |
|  | Yes -> Yes | 8263 | 202 | 69351.69 | 2.91 | 1.27 (1.11, 1.46) |  | 1.28 (1.11, 1.48) |  |

Supplementary Figure S1. Calibration plot of the final adjusted Cox model (Model 6) comparing predicted and observed probabilities for colorectal cancer incidence.
